# Genomic effects on advertisement call structure in diploid and triploid hybrid waterfrogs (Anura, Pelophylax esculentus)

**DOI:** 10.1186/1472-6785-13-47

**Published:** 2013-12-04

**Authors:** Alexandra Hoffmann, Heinz-Ulrich Reyer

**Affiliations:** 1 Institute of Evolutionary Biology and Environmental Studies, University of Zurich, Zurich, Switzerland

**Keywords:** Advertisement calls, Dosage effects, Geographic variation, Genetic distance, Ploidy effects

## Abstract

**Background:**

In anurans, differences in male mating calls have intensively been studied with respect to taxonomic classification, phylogeographic comparisons among different populations and sexual selection. Although overall successful, there is often much unexplained variation in these studies. Potential causes for such variation include differences among genotypes and breeding systems, as well as differences between populations. We investigated how these three factors affect call properties in male water frogs of *Pelophylax lessonae* (genotype LL), *P. ridibundus* (RR) and their interspecific hybrid *P. esculentus* which comes in diploid (LR) and triploid types (LLR, LRR).

**Results:**

We investigated five call parameters that all showed a genomic dosage effect, i.e. they either decreased or increased with the L/R ratio in the order LL-LLR-LR-LRR-RR. Not all parameters differentiated equally well between the five genotypes, but combined they provided a good separation. Two of the five call parameters were also affected by the breeding system. Calls of diploid LR males varied, depending on whether these males mated with one or both of the parental species (diploid systems) or triploid hybrids (mixed ploidy systems). With the exception of the northernmost mixed-ploidy population, call differences were not related to the geographic location of the population and they were not correlated with genetic distances in the R and L genomes.

**Conclusions:**

We found an influence of all three tested factors on call parameters, with the effect size decreasing from genotype through breeding system to geographic location of the population. Overall, results were in line with predictions from a dosage effect in L/R ratios, but in three call parameters all three hybrid types were more similar to one or the other parental species. Also calls of diploid hybrids varied between breeding systems in agreement with the sexual host required for successful reproduction. The lack of hybrid call differences in a mixed-ploidy population at the northern edge of the water frog distribution is likely to be associated with genetic particularities, including a) low genetic variability and/or b) a local loss of genes coding for genotype-dependent call differentiation under conditions where female discrimination between diploid and triploid males is not beneficial.

## Background

Acoustic communication in animals often mirrors selective forces that generate and maintain evolutionary change. In anurans, bioacoustic characteristics of male advertisement calls are important traits shaped by sexual selection and serve as signals for male quality and species recognition. Thus, anuran mating calls have been frequently used for studies of mate choice [1-4] but also for taxonomic purposes and phylogenetics (e.g. [8,9]). In several anuran taxa advertisement calls have helped in identifying cryptic species pairs [10,11] and separating interspecific hybrids from their parental species [12]. Nevertheless, some authors have cautioned against the use of male calls for frog identification because of considerable within-taxon variation and great overlap in call features among hybrid and parental taxa [13,14]. Major factors responsible for this call variation and overlap are differences in genotypes (1) breeding systems (2) and geographic and genetic distances (3).

1. **Genotypes:** An important genomic particularity that can alter phenotypic expression is polyploidization, which often comes along with hybridization and enables hybrids to overcome meiotic difficulties in order to successfully reproduce [15,16]. Studies on call structure in polyploid anuran taxa of hybrid origin have revealed a causal relationship between ploidy and advertisement call structure [17-19]. Empirical studies using artificially created autotriploid and natural allotriploid Hylid frogs have shown direct effects of polyploidy on triploid male advertisement call structure [20] and even parallel developing call preferences in triploid females [21]. According to the results from these studies, changes in triploid male advertisement calls were causally related to a polyploidy-induced increase in cell size. In addition, phenotypic traits of polyploids can be expected to correlate with the relative numbers of the two parental genomes in the hybrid individual (“dosage effect”). Such correlations are well-known in plants (reviewed by [22]). In water frogs, [23] have recently demonstrated this for some morphological characters, but the combined results from other studies on water frogs yield no general support for the idea that traits of hybrid water frogs are shaped by dosage effects (reviewed by [24]).

2. **Breeding systems:** Hybrids of different ploidies may further differ in call characteristics from their parental species and from each other as a result of various selection regimes, be it natural selection due to different acoustic environments or predator pressures [8,25-27], be it sexual selection arising from differences in mate choice preferences [28], or be it character displacement when different forms become reproductively isolated. Again, empirical support for such selection regimes is mixed. Some studies do find differences in advertisement calls and female preferences between polyploidy forms and their diploid relatives [29,30], whereas others do not [11,31,32]. This is likely to reflect different selections pressures on male advertisement calls and female choice and, hence, can be expected to differ with the breeding system.

3. **Geographic and genetic distances among populations:** Interspecific hybridization can result in persistent call alterations in hybrids, due to genetic or chromosomal interactions that can cause changes in the morphology of the laryngeal apparatus [31], the nervous system [33], and the contractile frequency of muscles [34]. Given that hybridization is not uncommon in amphibians, it is likely to occur multiply across the area where two species overlap. When there is geographic variation in genetic, morphological, physiological and acoustical traits within the two parental species (as shown by [12,35,36]), hybrids from different ancestral populations can be expected to produce different calls. On the other hand, genetic isolation by distance could cause populations of common origin to drift apart which will result in differences in several phenotypic traits, including advertisement call patterns [36,37].

### The study system

**Figure 1:**
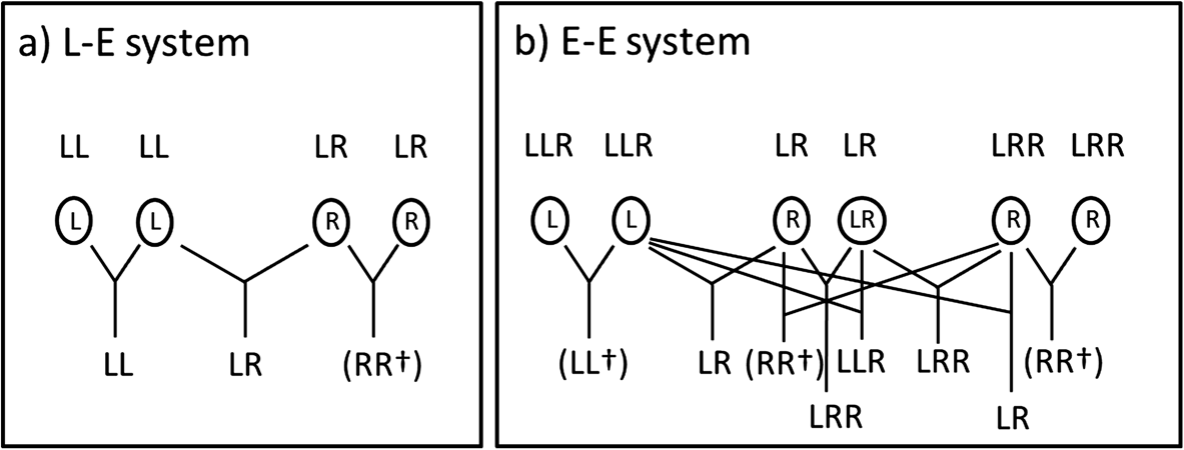
**Overview of adult genotypes, gametes types (in circles) and resulting offspring.** LL = *P. lessonae*, LR, LLR and LRR = *P. esculentus*. **a)** the L-E system, where only diploid hybrids are produced, and non-hybrid genotypes from matings between hybrids typically die prematurely. **b)** the E-E system, where all three hybrid types can cross, but only hybrid genotypes survive to reproductive maturity. In the L-E system, the R genome is never recombined, and L genomes are provided by *P. lessonae*. In the E-E system, three types of gametes are produced by hybrids and both L and R genomes regularly undergo recombination when they are present in double copy in triploid hybrids (Christiansen 2009).

An excellent model organism for studying how these three factors influence advertisement calls is the Edible Frog *Pelophylax esculentus* (called *Rana esculenta* until [38])*,* the most widespread and successful anuran hybrid in Europe. Its geographic distribution ranges from about 44° latitude in the south (southern France to northern Bulgaria) to 60° in the north (southwest Sweden to Baltic countries) and from the French Atlantic coast in the west to western Russia in the east (for details see Figure 1.18 in [24]). The hybrid originally arose (and still arises) from interspecific matings between *P. lessonae* (Pool Frog) and *P. ridibundus* (Marsh Frog). The hybrid has abandoned the normal inheritance pattern of chromosomes and developed alternative ways of gamete production that circumvent incompatibilities between the parental genomes during meiosis. The typical and most widespread way is hybridogenetic (= hemiclonal) reproduction, meaning that one of the parental genomes is excluded prior to meiosis and the other one clonally transmitted to haploid eggs and sperm, respectively [39,40]. Hybridity and the diploid state are restored by back-crossing with the parental species whose genome was excluded. Depending on the specific genetic interactions between the hybrid and the parental species, three major breeding systems can be distinguished: the L-E-, R-E- and E-E-system [24,41-43]. In the so-called L-E-breeding system (referring to the Latin names *lessonae* and *esculentus*), the excluded genome is that of *P. lessonae*, whereas in the R-E-breeding system (for *ridibundus-esculentus*), the *P. ridibundus* genome is excluded. In both cases, the hybrid has to live in sympatry and mate with one the respective parental species to regain the previously eliminated genome for its offspring. In these two breeding systems, all individuals are diploid and hybrids can only produce viable offspring when mating with the parental species, since crosses between two hybrids are usually lethal (Figure 1a). Hence, the hybrid is a sexual parasite that needs a parental species as a sexual host for successful reproduction. At least for the L-E system this is also reflected in the mating behavior: both theoretical models and mate choice experiments have shown that diploid hybrid females (LR) should - and do - prefer LL males over their own [6,44,45].

In some populations this hybridogenetic mode of reproduction that is typical for diploid systems is modified in a way that hybrids have become entirely independent from the need to backcross with a parental species. As a result viable all-hybrid populations can exist. Such populations are concentrated in areas around the Baltic Sea, but also occur in some other areas of Europe [24,46-52]. The explanation for the existence of such all-hybrid populations lies in the coexistence of diploid (LR) and triploid (LLR, LRR) animals in the same population [53]. The best-described diploid-triploid all-hybrid population system is the so-called E-E-breeding system (in reference to successful *esculentus-esculentus* pairings). In the typical and most widespread case, diploid hybrids (usually females) produce diploid gametes that result in viable triploid offspring when they fuse with haploid gametes (Figure 1b). These haploid gametes can either be provided by diploids (usually males) through the hybridogenetic mechanism described above or by triploids of both sexes that exclude the single-copy genome (R in LLR and L in LRR) before they recombine the two remaining homospecific genome copies (LL and RR, respectively) during a normal meiosis [54,55]. Thus, in these mixed ploidy populations triploid hybrids adopt the role as sexual hosts for the diploid hybrids that the parental species have in diploid L-E and R-E-systems. In these all-hybrid systems, occasional fusion of two diploid gametes results in tetraploids, but these appear to be extremely rare in natural populations and have not yet been investigated in terms of their reproductive mode [54,56]. Triploid forms, on the other hand, are widespread, and their reproductive patterns have been studied intensely for a number of decades [54,56-64], including the mating behavior which, in contrast to the diploid L-E-system, seems to be random. For E-E-systems both theoretical models and empirical studies have shown that no preference should exist in hybrid females; and apparently it does not [65,66]. Within the parental species’ distribution ranges, diploid-triploid *P. esculentus* populations often co-exist and interbreed with parental genotypes, thus forming mixed populations [24].

With its various hybrid genotypes, different breeding systems and wide geographic distribution, *P. esculentus* provides all the variation that is required for testing in the same organism how genotypes, breeding system and geography influence variation in male advertisement calls. This is what we attempted in this study, starting with predictions from the following three not mutually exclusive hypotheses:

1. **Genotype hypothesis:** With L/R genome ratios differing among genotypes, dosage effects predict a directional increase (or decrease) in call parameter values in the order LL–LLR-LR-LRR-RR (i.e. 1.00-0.67-0.50-0.33-0.00).

2. **Breeding system hypothesis:** As hybrid females in diploid breeding systems must choose partners of a parental species for successful reproduction, whereas those in all-hybrid breeding systems with mixed ploidy should not have a preference, we expect different selections pressures on male advertisement calls. Hence, the selection hypothesis predicts that calls of the same hybrid genotypes will differ with the breeding system.

3. **Geographic hypothesis:** Given the wide distribution range of *P. esculentus* across Europe*,* the geographic hypothesis predicts that hybrids of the same genotype from far apart populations will differ in their advertisement calls. These differences could be due to their supposed origin from multiple primary hybridization events between *P. lessonae* and *P. ridibundus* from different populations and/or a common origin followed by drift [36,37].

To test the predictions from these three hypotheses, we compared call parameter variation between hybrids of different genotypes (1), from different breeding systems (2), and from far geographically apart populations (3). Further, we examined advertisement call variation on a population level against genetic and geographic distance between populations. To our knowledge, this is the first study on bioacoustic differences that includes both hybridogenetic and sexual populations of the same anuran hybrid complex and is able to compare different genotypes and hybrids of different ploidies over an extensive geographic scale and in a population genetic context. Previous studies have provided extensive data on the genetic and inheritance patterns in populations with different ploidies, but empirical data on phenotypic manifestations in triploid versus diploid water frogs, or in recombining versus hybridogenetically reproducing hybrids are restricted to cell planimetry and body morphology [23,67-71]. Where vocalization and other behaviors were investigated and found to vary within hybrid lineages [72-74], these studies were mostly restricted to mixed populations of diploid *P. esculentus* and one or both of its parental species. The same is true for studies using male advertisement calls for distinguishing between water frog species and populations [12,36].

## Methods

### Selection of populations

**Figure 2:**
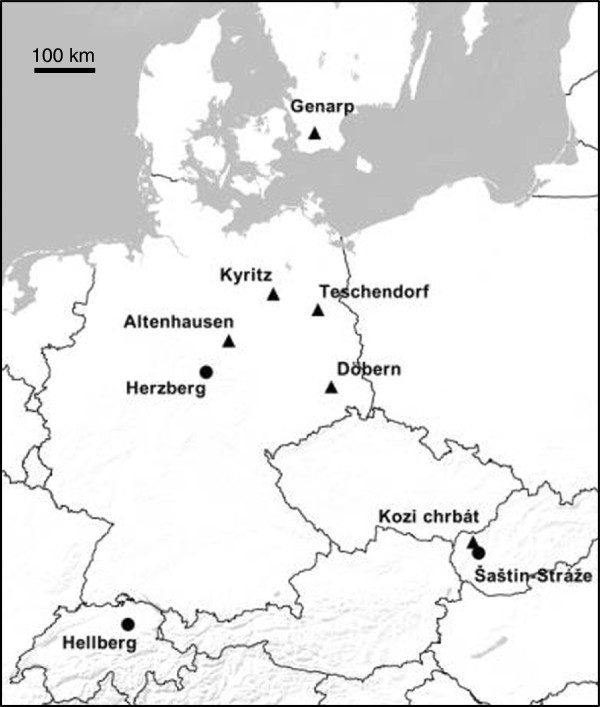
**Map showing geographical locations of recorded populations.** Dots = diploid populations with diploid hybrids and one or both parental species; triangles = mixed ploidy populations with mainly diploid and triploid hybrids, sometimes in sympatry with very few *P. lessonae*. For absolute numbers see Table 1.

In line with our three hypotheses, we recorded advertisement calls in nine populations with varying combinations of genotypes (hypothesis 1), breeding systems (hypothesis 2) and geographic distances (hypothesis 3). A map is shown in Figure 2. Choice of the study populations was based on relevant information from earlier studies [67,75], (Reyer unpublished data [76,77]). In terms of the breeding system, we differentiated between two hybrid systems, a) L-E and L-E-R populations, where diploid *P. esculentus* occur in sympatry with *P. lessonae* and/or *P. ridibundus*, produce haploid R gametes and backcross with *P. lessonae* (rarely *P. ridibundus*) and b) E-E and L-E-E populations, where some individuals form diploid gametes, and both diploid and polyploid hybrids either reproduce without backcrossing or by only occasionally mating with their parental species (usually *P. lessonae*). For ease of expression, we will refer to the populations described under a) as “diploid systems” (only haploid gametes and diploid hybrids are produced), and to the populations described under b) as “mixed ploidy systems” (due to the occurrence of diploid gametes, both diploid and polyploid hybrids can be produced).

In the three geographically distant diploid systems, diploid *P. esculentus* are sympatric with one parental species (*P. lessonae,* Hellberg) or both (*P. lessonae* and *P. ridibundus,* Herzberg and Šaštin-Stráže) (Table 1). Among the six populations of mixed ploidy, three contained diploid and triploid hybrids only, while in the other three (Altenhausen, Teschendorf and Kyritz) very few *P. lessonae* males and females were found (6 out of 147 individuals in total). In five of the six mixed ploidy populations all three hybrid genotypes (LR, LLR, LRR) were found at least in one sex; in the sixth population (Kozi chrbát, Western Slovakia) only LLR males and LR females were caught on this and one other occasion. Triploid LRR hybrids and LLR females are not known from this region, but LR males probably exist (Pruvost et al., subm.). In Teschendorf, only LR and LLR males have been found, although LL males are known to be present in this population (J. Plötner, pers. comm.).

**Table 1:** Genotype distribution, number of genetic samples and genome-specific gene diversity He in diploid an and mixed ploidy breeding systems

**Population**	**Ploidy types**	**Counts per type**		**N genetic samples (all types)**		**He (all types)**	
Diploid systems		♂	♀	L	R	L	R
Hellberg	LL	11	14	14	3	0.67	0.06
	LR	3	6				
Herzberg	LL	9	2	21	22	0.45	0.40
	LR	12	3				
	RR	10	-				
Šastín-Štraze	LL	4	1	48	49	0.5	0.45
	LR	27	16				
	RR	2	4				
Mixed ploidy systems							
Altenhausen	LL	3	1	29	26	0.36	0.53
	LLR	17	5				
	LR	8	1				
	LRR	1	-				
Teschendorf	LL	-	1	34	33	0.37	0.35
	LLR	21	2				
	LR	2	4				
	LRR	-	1				
Kyritz	LL	1	-	81	80	0.34	0.40
	LLR	9	4				
	LR	18	20				
	LRR	10	19				
Genarp	LLR	14	4	175	175	0.15	0.12
	LR	106	24				
	LRR	3	24				
Döbern	LLR	33	3	149	149	0.16	0.30
	LR	24	23				
	LRR	48	17				
Kozi chbát	LLR	89	-	105	105	0.29	0.39
	LR	-	16				

Gene diversity He is based on allele frequencies in L and R genomes, corrected for sample size (Nei 1978).

Due to high variation in abundance, not all occurring types could be recorded within each population, and not in equally high numbers. In mixed-ploidy systems this was especially true for LRR males which were extremely rare in most populations, with the exception of Döbern (Table 1). In Altenhausen and Kyritz, single LL males have been observed but could not be recorded.

### Field work

In early summer of 2009, 2010 and 2011 we collected field recordings of male advertisement calls from the selected populations (Table 2). In Döbern and Genarp, we individually marked males using elastic and degradable waist-bands with clearly legible numbers to make them identifiable during repeated recordings. In other ponds we recorded calling males first and captured them directly afterwards for body size measurements and genetic identification. Additionally, some males were brought to Zurich and recorded in semi-natural outdoor ponds. During all recordings, focal males were recorded from a distance of 50–100 cm with microphones attached to a 1.5m bamboo stick and a hand-held digital recorder (Zoom H4n). We used a set of two mono-channel condensor microphones (AKG C417PP), one directed at the frog and the other attached to the observer. The two channels enabled separate recordings of frog calls and observer comments on the caller’s identity, allowing later distinction of simultaneous calls by several males in dense choruses. We recorded water temperatures just below surface level close to calling individuals. Most recordings were taken during peak calling activity, which usually takes place at water temperatures ranging from 17.5-22°C [78]. In our study, mean water temperature was 21.7 ± 2.0°C (S.D., range 16.0-28.7 C).

**Table 2:** Population systems, number of recordings per genotype type and geographic coordinates of study populations

**Breeding system**	**Recordings per genotype**					**Population**	**Coordinates**	
	**LL**	**LLR**	**LR**	**LRR**	**RR**			
Diploid	3		3			Hellberg (CH)	'47°17′45.72″N'	'8°48′48.38″E
	6		5		6	Herzberg (D)	51°37′36.66″N	'10°21′15.06″E
	1		4		2	Šastín-Štraze (SK)	'48°37′54.61″N	'17°8′40.38″E
Mixed Ploidy		6	8			Altenhausen (D)	52°16′40.00″N	11°15′15.00″E
		6	2			Teschendorf (D)	52°51′53.03″N	13° 8′40.38″E
		4	6	4		Kyritz (D)	52°54′07.08″N	12°19′15.50″E
		5	7	2		Genarp (SE)	55°36′34.00″N	13°23′19.00″E
		6	6	6		Döbern (D)	51°36′38.22″N	14°36′15.60″E
		6				Kozi chrbát (SK)	'48°37′53.58″N	17°17′41.28″E
Total (n = 104)	10	33	41	12	8			

Recording numbers equal sample sizes per genotype for all analyses including call parameters.

### Advertisement calls

**Figure 3:**
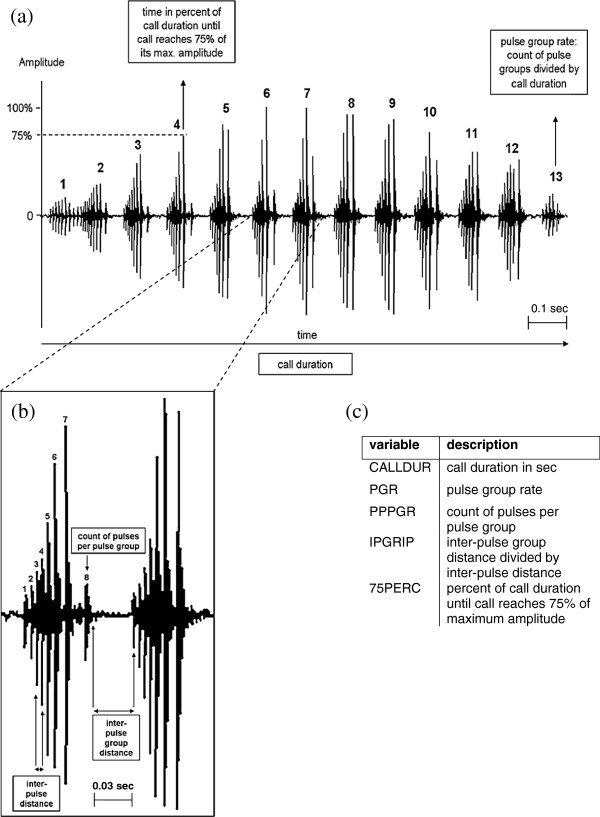
**Measurements taken for call variables. (a)** measurements on *P. esculentus* call (LR type), **(b)** single pulse group and pulse measurements, and **(c)** list of derived variables used for subsequent analyses.

Water frog advertisement calls comprise a number of single pulses, which are bundled groups of varying distinction (Figure 3a). We defined the parameter pulse group (PGR) as a visible structure in the pulse sequence of a call. This structure can be either temporal (i.e. through long intervals between groups of condensed pulses) or energetic (i.e. through regular differences in amplitude that cause a visible pattern, although between-pulse group intervals can be short). For characterizing the temporal quality of the call, we used the following parameters: the entire length of the call (CALLDUR), the rate of pulse groups divided by the length of the call (PGR), the number of pulses per pulse group (PPPGR) and the ratio of inter-pulse group intervals to inter-pulse distance (IPGRIP). The latter describes the shape or “condensation” of pulse groups along the time axis. Energetic properties were expressed by the percentage of call duration that passes until the call energy rises from 10% to 75% of its maximum amplitude (75PERC). Although the rise from 10% to 90% is a more conventional measure (C. Gerhardt, pers. comm.), we used the smaller range, because the two measurements are strongly correlated and the 10%-75% measurement showed fewer outliers and more normal data distribution. Variables CALLDUR, PGR and 75PERC yielded one value per call, while the pulse-group-based parameters PPPGR and IPGRIP were averaged over 4 measurements taken at regular intervals over the entire call. We generally measured and averaged 6 calls of good recording quality per individual. For 20% (21/104) of the males, averages could only be taken from 3–5 calls per individual.

Calls were cut and edited using the program ACOUSTICA 4.0. Call parameters were selected and measured in the program Avisoft SASPro. For comparative analyses between populations and genotypes we used the five above described temporal and energetic call parameters, which have been successfully applied to discriminate among anuran calls in other studies [20,29]. We did not include spectral properties of the calls, since they can be strongly affected by calling context [79], which is difficult to quantify and, hence, was not recorded.

### Population composition and genetics

Since the number of sound-recorded individuals per population was too low to calculate meaningful genotype ratios and population genetic parameters, we included additional samples from both males and females for most populations; these were collected at the same or a previous time for a different study with the aim of identifying genotype and sex ratios at these sites (see Table 1). Frogs were collected by hand or with a net, and a tissue sample (toe clip) was taken upon capture. To specify the genotype we used species- and genome dosage-specific allelic information from microsatellite markers. For this, DNA from ethanol-stored toe clips was extracted using the Qiagen BioSprint 96 DNA Blood Kit and the corresponding tissue extraction protocol. With each tissue sample two multiplex PCRs were conducted with 9 primer pairs each. Protocols of DNA purification, extraction and PCR are described elsewhere [58,80]. A list of primers and sequences is given in the supplementary material (Additional file 1). Singleplex PCRs were also run to check the results of some primers. PCR products were run on an ABI 3730 Avant capillary sequencer (Applied Biosystems, Life Technologies Corporation, Carlsbad, CA) with internal size standard (GeneScan-500 LIZ). Two loci were problematic due to amplification problems across all populations (one due to unambiguous allele specificity) and therefore the primers were excluded. Among the remaining 16 primers, 4 amplified the L, 8 the R and 4 both genomes (for details see Additional file 1). Those with markers for both genomes showed dosage-effects that were used to distinguish between LLR, LR and LRR by the relative density of the amplified species-specific alleles [80,81]. In total, the 16 primers amplified 13 loci for each genome.

Alleles were scored in the program Genemapper (Applied Biosystems 2004, Genemapper vers. 3.7.). Most alleles could be assigned unambiguously to either the L or the R genome, and individuals showed a clear “consensus genotype”, i.e. the same genotype for each of the used microsatellite markers. We checked the data set for existing null alleles separately for the L and R genome. Null alleles are unmasked and can be easily detected in the hemizygous state of LR hybrids and in single genome copies of triploids (R in LLR, L in LRR) (for details see [55]). Correspondingly, null alleles can potentially be masked in homozygous individuals of the parental species and triploid hybrids carrying two copies of the genome in question. Populations and loci where no unmasked null alleles were detected we considered null-allele-free. In few cases of null alleles (< 2 cases per population), the individual was excluded from the data set. At two different loci (RlCA1b6 and Re1Caga10), more than 2 cases of unmasked null alleles were found in one and two populations, respectively. This means that a relevant number of undetected null alleles might exist in these populations and could potentially bias population genetic estimates. At locus RlCA1b6, all individuals of the concerned population (Altenhausen) were hemizygous (either LR or LLR with unmasked null in the R genome). Thus, the null allele could always be detected and was therefore coded as a real allele. However, at locus Re1Caga10 both hemi- and homozygous individuals of two populations (Šaštin-Stráže and Teschendorf) were affected (LL and LR, null occurring in the L genome). Therefore, the entire locus was recoded as missing data and not used for analysis.

Genetic variation was expressed by gene diversity (H_e_), which is gene variability corrected for sample size, and Nei’s D [82], both calculated in the program SPAGeDi version 1.3. SPAGeDi accepts haploid and diploid individuals in the same analysis under the assumption that the two genomes in diploid individuals recombine. The program also requires individuals to have only one genome type, which is violated in hybrids. To circumvent this problem, we separated the L and R genome data into two different input files and analyzed them with a method, that has been successfully employed by other authors [46,52,54,58,75]: LR hybrids were treated as haploid for both genomes, LLR and LRR hybrids were treated as haploid for the single haploid and diploid for the double genome, and the parental species (LL and RR) were treated as diploid for the L and R genome, respectively.

### Statistical analyses

For testing whether and how the five call parameters mentioned above differ among genotypes and localities we performed a separate GLM for each parameter. Since several call properties are influenced by body size and water temperature [31,83,84], we included these variables as covariates. All GLMs were perfomed in a stepwise manner with backward elimination; starting with the full set of predictive variables, we successively dropped those with a probability > 0.05. Effect size, i.e. the strength of the association between a significant predictive variable and the dependent variable, was calculated as η^2^ = SS_effect_/SS_total_, where η^2^ is the proportion of the effect variance (SS_effect_) to the total variance (SS_total_). Conventional critical values for small, medium and large effect sizes are 0.10, 0.25 and 0.40, respectively [85].

For investigating how genotypes differed in their overall mating call structure, we performed a discriminant analysis on the set of all five call parameters with genotype as the separating factor. Based on the results from this analysis, we averaged the first two canonical scores for each genotype from each population. From these averages we created a matrix of pairwise Euclidean distances as an index of average call dissimilarity (also referred to as call distance in the following). This measure was used to structure the different genotypes of all populations in a hierarchical cluster analysis implementing the group average linkage type method [86]. For a subsequent two-sample comparison between two different breeding systems, we used a multivariate Hotelling’s T-test with 10000 random permutations, since the low number of sample sizes was inadequate for a discriminant analysis. To perform multiple correlation analyses we created different pairwise distance matrices.

To address the question whether call variation patterns were correlated with geographic distance and/or genetic distance (a link to be expected under isolation by distance), we performed Mantel tests, based on genotype-specific subsets of call distance and genetic distance data. For a matrix of geographic distances, Euclidean distances between sampling sites were calculated from GPS coordinates into inter-population distance data (km) using an online GPS Latitude and Longitude Distance Calculator (http://www.csgnetwork.com/gpsdistcalc.html). For genetic comparisons, pairwise matrices of Nei’s D were created for the L and R genome separately, based on the full sample size (including all genotypes that carry the genome in question) for each population, using the program SPAGeDi. Pairwise distance matrices of geography, call dissimilarity and Nei’s D values were then correlated with simple and partial Mantel tests in the program zt 1.2 [87] with 1000 permutations. Simple Mantel tests were performed to find correlational relationships between two pairwise distance matrices, and partial Mantel tests were used to control for potential covariation by a third distance variable matrix [88]. Earlier studies on polyploid water frog populations had indicated that such covariance might occur between genetic distance and geographic distance and potentially influence call distance. Significance tests were computed by running 1000 iterations of the data set. To avoid mixing populations of different reproductive modes, we restricted these analyses to a subset of populations containing both LR and LLR individuals. Unfortunately, the number of populations containing only diploid hybrids and those containing LRR hybrids were too low to perform Mantel tests for these groups.

Unless otherwise stated, statistics were performed in the programs NCSS [89] and SYSTAT 11 [90].

## Results

### Differences in single call parameters

**Figure 4:**
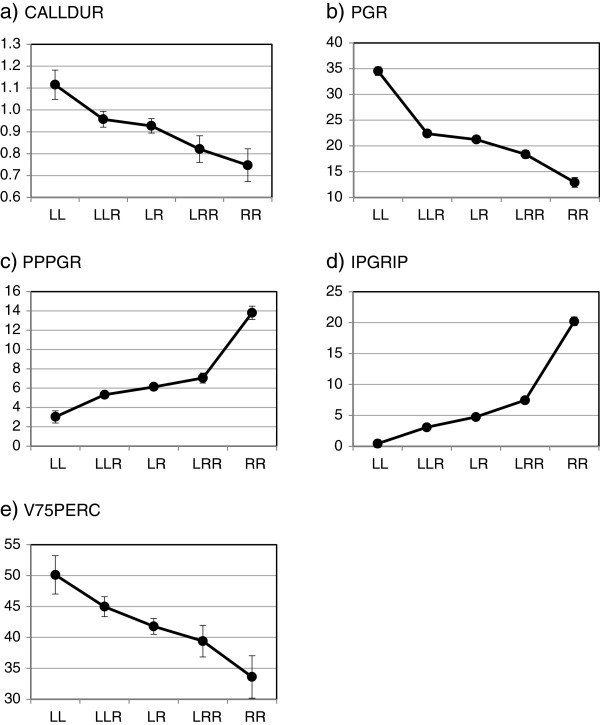
**Means (± 1 S.E.) of the five call parameters (panels a-e) defined in Figure **3**for *P. lessonae *(LL), *P. ridibundus *(RR) and their interspecific diploid (LR) and triploid (LLR, LRR) hybrids *P. esculentus*.** Sample sizes are given in Table 2.

Variation in each of the five call parameters was investigated in relation to two categories (genotype and population) and two covariates (water temperature during calling and male body size) by means of GLMs. Results are shown in Table 3 and Figure 4. Among the covariates, body size had no effect on any of the five call parameters and temperature influenced only three of them: CALLDUR decreased and PGR and IPPGRP increased with increasing temperatures. However, although significant, the size of the temperature effect was low for all three parameters explaining only 12%, 7% and 1% of the variation, respectively (see η^2^ values in Table 3).

**Table 3:** Results from GLMs testing for the effects of two categories (genotype and population) and two covariates (water temperature and male body size) on five call parameters

**Call parameter**	**Statistics**	**Genotype**	**Population**	**Temperature**	**Body size**
CALLDUR	F	4.348		15.398	
	P	0.003		<0.001	
	η^2^	0.132		0.117	
PGR	F	84.221		35.314	
	P	<0.001		<0.001	
	η^2^	0.715		0.075	
PPPGR	F	54.368	6.532		
	P	<0.001	<0.001		
	η^2^	0.601	0.144		
IPGRIP	F	207.650	4.616	5.305	
	P	<0.001	<0.001	0.024	
	η^2^	0.862	0.038	0.006	
75PERC	F	7.697			
	P	<0.001			
	η^2^	0.237			

Shown are F-ratios, P values and effect sizes (η^2^) for significant relationships.

The strongest and most consistent effect on call parameters was exerted by genotype, which showed a significant influence on all five call parameters. Differences among genotypes were always directional, with means either increasing (PPPGR, IPGRIP) or decreasing (CALLDUR, PGR, 75PERC) in the order LL, LLR, LR. LRR, RR, i.e. with the ratio of L/R genomes (1.00, 0.67, 0.50, 0.33, 0.00). LL calls were of longer duration, higher pulse group rate and reached 75% of the maximum amplitude later than RR calls, whereas the number of pulses per group and the ratio of inter-pulse group intervals to inter-pulse distance were higher for RR than for LL. For CALLDUR and 75PERC, there was an almost linear decrease from LL through hybrids to RR, whereas for PGR hybrid means were slightly closer to RR and for IPGRIP and PPPGR closer to LL.

Bonferroni posthoc tests revealed the following significant pairwise differences: all five genotypes differed from each other in IPGRIP and all but LLR and LR also in PGR and PPPGR. For the remaining two parameters only the most extreme pairs differed significantly: LL from LRR and RR in CALLDUR and LL – by trend also LLR (P = 0.075) - from RR in V75PERC. Together with the fact that IPGRIP also had the highest effect size (0.86), these results indicate that this variable differentiated best between genotypes.

For two call parameters (PPPGR and IPGRIP) there were also significant population effects (Table 3). Posthoc pairwise comparisons showed that it was mainly the northernmost population of Genarp that differed from the rest. Here, mean values for both call parameters were lower than in the other populations. Moreover, a separate analysis for this population revealed no significant differences between the three hybrid types (LLR, LR, LRR) for any of the five call parameters (all F_2,9_ ≤ 0.693, all P ≥ 0.525).

### Overall call differences

**Figure 5:**
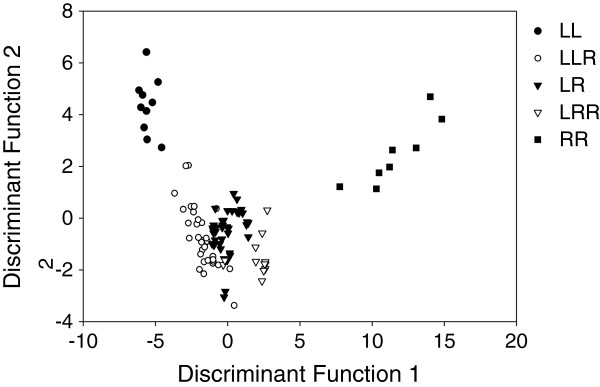
**Discriminant functions plot of water frog calls.** Canonical scores 1 and 2 were calculated from the combined parameters CALLDUR, PGR, PPPGR, IPGRIP and 75PERC.

For analyzing the overall call differences among the two parental and three hybrid taxa, we subjected all five call parameters to a discriminant analysis with genotype as the separating category. We obtained four discriminant functions, but only the first two functions were significant (Table 4a). Together they accounted for more than 99% of the total dispersion. Function 1 had the strongest influence on data separation (83.4% of total dispersion). Correlations of function 1 with the five call parameters mirrored the results from preceding univariate analyses. Function 1 was positively correlated with IPGRIP (*r* = 0.77) and PPPGR (*r* = 0.37), and negatively correlated with PGR (*r* = -0.43), CALLDUR (*r* = -0.13) and 75PERC (*r* = - 0.13). Correlations between parameters and discriminant function 2, which accounted for 16% of total dispersion, were positive for all call parameters (IPGRIP: *r* = 0.43; PPPGR: *r* = 0.21; CALLDUR: *r* = 0.18; 75PERC: *r* = 0.12), and strongest for PGR (*r* = 0.55). Again, IPGRIP was the call variable with the highest power of discrimination for genotype as a grouping factor. A scatter plot of the first two canonical functions is shown in Figure 5, illustrating that the discriminant analysis results based on our five parameters resulted in little overlap between genotypes.

**Table 4:** Results of multivariate discriminant analysis with genotype as discriminating factor

**(a) Canonical variate analysis for discriminant functions.**							
**Function**	**Eigenvalue**	**% of total dispersion**	**Canonical correlation**	**Wilk’s lambda**	**F**	**df**	**P**
1	15.52	83.4	0.969	0.013	41.9	20	< 0.0001
2	2.98	16.0	0.865	0.225	16.0	12	< 0.0001
3	0.09	0.5	0.299	0.898	1.8	6	0.11
4	0.01	0.1	0.114	0.986	0.6	2	0.52
**(b) Classification matrix**							
			**Predicted**			**% correctly classified**	
Actual	LL	LLR	LR	LRR	RR		
LL	10	0	0	0	0	100.0	
LLR	0	29	3	1	0	87.9	
LR	0	3	35	3	0	85.4	
LRR	0	0	2	10	0	83.3	
RR	0	0	0	0	8	100.0	

Classification of individuals was highly successful, with an overall 88.5% (92 out of 104 individuals) classified to the correct genotype (Table 4b). One hundred percent correct classification was achieved for the two parental types RR and LL, whereas correct classification of hybrids was only between 80 and 90%. LLR hybrids were three times misclassified as LR and once as LRR, and LRR were twice misclassified as LR. Diploid LR hybrids were six times misclassified as triploids, three times each as LLR and LRR.

### Call structure in relation to breeding systems

Although with 85% classification success for diploid hybrids was fairly high, it was not perfect. We therefor tested whether some of the unexplained variation might be caused by the affiliation of diploid hybrids with one or the other of the two earlier defined breeding systems, i.e. whether LR calls differ between diploid populations, where successful hybrid reproduction requires preference for and mating with individuals of a parental species, and mixed ploidy systems which can be maintained by random hybrid x hybrid matings. Since the overall sample size of LR hybrid calls was too low to perform a reliable discriminant analysis between the two breeding systems, we performed a multivariate Hotelling’s T-test with 10000 random permutations instead. The test results show a highly significant difference between diploid and mixed ploidy systems when combining all call characteristics for LR hybrids (All variables combined: Hotelling’s T^2^_5,31_ = 189.05, P = 0.0001). In subsequent parametric T-tests using all five variables individually, only two variables turned out discriminative: PPPGR (T_2,31_ = 3.39, P = 0.002) and IPGRIP (T_2,31_ = 8.31, P < 0.0001). For both variables, mean values of LR calls from diploid systems were less similar to *P. lessonae* than those from mixed ploidy systems. Although not significant, there was an indication that this shift towards the *P. ridibundus* pattern was mainly in the populations of Herzberg and Šaštin-Stráže, where both parental species occur, whereas in Hellberg where *P. ridibundus* is absent values for LR were not different from the overall averages shown in Figure 4c and d (Herzberg: PPPGR = 8.86 ± 1.16, IPGRIP = 10.47 ± 2.04; Šaštin-Stráže: PPPGR = 7.71 ± 1.80, IPGRIP = 9.17 ± 3.14; Hellberg: PPPGR = 5.72 ± 1.94, IPGRIP = 4.02 ± 3.43; means ± SE). The means of the remaining variables did not differ between the two breeding systems (CALLDUR: T_2,31_ = 1.59, P = 0.12; PGR: T_2,31_ = 0.67, P = 0.51; 75PERC: T_2,31_ = 0.96, P = 0.34).

### Call structure in relation geographic and genetic distances between populations

**Figure 6:**
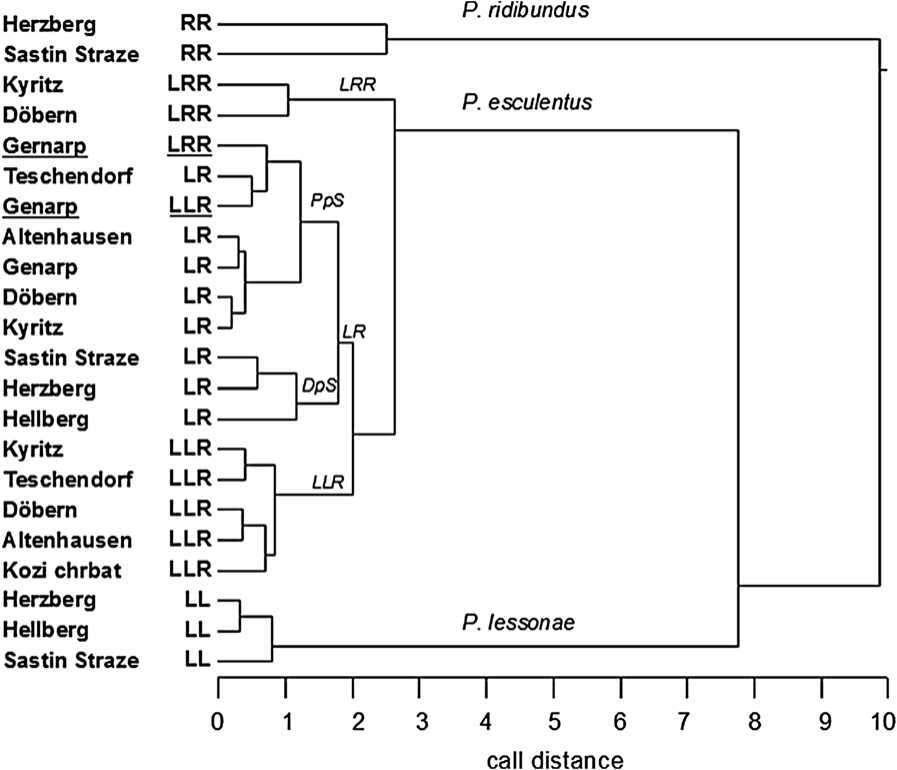
**Cluster dendrogram calculated from Euclidean call distances (based on discriminant analysis scores) between genotypes from different populatio ns.** Given in italics are species names, *P. esculentus* ploidy types (LRR, LR, LLR), and population systems within the LR group (Pps = polyploid system, DpS = diploid system). Two groups (LLR and LRR from the most northern population of Genarp) are underlined to indicate their exceptional behavior within the overall pattern of genotype-specific clustering.

A hierarchical cluster analysis based on Euclidian distances between the two significant discriminant functions was performed to examine and visualize call similarities, respectively distances, in relation to population, i.e. geographic location. In the resulting dendrogram (Figure 6), hybrid *P. esculentus*, parental *P. ridibundus* (RR) and *P. lessonae* (LL) formed three main clusters. RR populations showed higher dissimilarity among themselves than LL populations, which were positioned closer to the hybrid than to the RR cluster. Within the hybrid cluster, LR, LLR and LRR formed separate and largely homogenous groups. These were independent from the population of origin, thus showing clear genotype-specific separation. Hence, call similarities are higher between same-genotype groups from different populations than between different genotypes from the same population. The only exception from this pattern was found in calls from the northernmost mixed ploidy population Genarp (southern Sweden). Here, calls of diploid and triploid frog types were nested in the larger LR cluster. Thus, LLR and LRR calls were more similar to each other and to sympatric LR calls than to calls of the corresponding triploids from other populations. This is consistent with the above described results from the univariate analyses of differences in single call parameters.

For a direct comparison of genetic diversity between diploid and mixed ploidy systems we used gene diversity corrected for sample size (Nei 1978) for the L genome (He_L_) and R genome (He_R_), respectively. Values of He for each marker were averaged for each genome. Mean gene diversity in the L genome (He_L_) was higher in diploid systems than in mixed ploidy ones (T_2,9_ = 3.21, P = 0.01), but the two systems did not differ in gene diversity in the R genome (He_R_; T_2,9_ = -0.30, P = 0.77). However, it should be considered that the value for He_R_ of one diploid population (Hellberg) was calculated from a very low sample size of individuals carrying an R genome (Table 1). Therefore, results for this population should be interpreted with caution.

Under the assumption that phenotypic data like differences in call characteristics should not influence isolation-by-distance in either genome, we performed simple Mantel tests between geographic distance and genetic distance for both genomes without controlling for a third matrix. These tests yielded no significant correlation between geographic distance and genetic distance (*R genome*: r = 0.33, P = 0.22; *L genome*: r = 0.02, P = 0.48). Subsequently, we tested whether call distances between populations within the same genotype were correlated to genetic and geographic distance by performing partial Mantel tests. These tests included call distance as a third matrix and alternatingly controlled for genetic and geographic distance, since these variables might still interact in their influence on call differences. In both LR and LLR hybrids, call similarity and genetic distance in the R genome did not correlate significantly after controlling for covariance by geographic distance (*R genome:* LR: partial r = 0.50, P = 0.2, LLR: partial r = -0.57, P = 0.14; *L genome:* LR: partial r = 0.10, P = 0.41). In the reverse tests, when controlling for genetic distance, we found no significant correlations among LR hybrids (*R genome:* LR: partial r = 0.08, P = 0.5; *L genome:* partial r = 0.09, P = 0.47). Among LLR hybrids, we did find some – yet non-significant – indication for a correlation between call distance and geographic distance when controlling for the R genome (partial r = 0.75, P = 0.10) and for the L genome (partial r = 0.63, P = 0.13).

Thus, there was no indication for a pattern of genetic isolation by geographic distance in the L and R genome, nor did call differences correlate with geographic or genetic distance.

## Discussion

### Genotype differences in male advertisement calls

In our study on male advertisement calls from nine *P. esculentus* populations across a broad geographic scale, we found significant genotype effects on all five call parameters that we considered. The strength of the genotype effects, however, varied among the call parameters. Effect sizes were small to medium for call duration (CALLDUR) and duration until 75% of the maximum amplitude was reached (V75PERC); large effect sizes were found in pulse group rate (PGR), the number of pulses per pulse group (PPPGR) and the condensation of pulse groups within the call (IPGRIP). These results from the univariate analyses of single call parameters were fully supported when all call parameters were combined in a multivariate analysis (Figure 5). Again, genotype discriminated very well between call properties, with 88.5% of all individuals correctly assigned. Triploid hybrids (LLR and LRR) scored closer to diploid hybrids (LR) than to their double-genome parental species, and were wrongly assigned only to other hybrids, but never to parental genotypes. A cluster analysis confirmed that, overall, call similarity was a strong population-independent separator among genotypes (with one exception, see below). The best separating parameter was IPGRIP. In the univariate analysis it had the largest effect size Table 3) and in the multivariate analysis the highest correlation (r = 0.77) with discriminant function 1. This function did not only clearly differentiate between the two parental species but also fairly well between the three hybrid types (Figure 5).

Earlier studies, partly using different call properties than we did, have already shown a robust differentiation between diploid *P. esculentus* and its parental species [12]. Our findings support this for additional call parameters, but – more importantly – they also reveal a differentiation between syntopic triploid and diploid hybrids for most mixed ploidy populations we sampled. For all five call parameters (Figure 4a-e) and for the combined data set (Figure 5) values either decreased or increased in the order LL-LLR-LR-LRR-RR, i.e. with L/R genome ratios of 1.00-0.67-0.50-0.33-0.00. This is in full agreement with expectations from a genomic dosage effect.

So far, evidence for a dosage effect on water frog traits is mixed (reviewed by [24]). In a recent study comparing morphological differences between LLR, LR and LRR hybrids and their LL and RR parental species, [23] found that differentiation in morphological indices are directional in the order LL-LLR-LR-LRR-RR, but the influence of the L haplotype was greater than the influence of the R haplotype. Thus, all hybrid types (including LRR) were morphologically closer to *P. lessonae* than to *P. ridibundus*. Conversely, [91] found that in triploid LLR hybrids most (but not all) morphological, ecological and biochemical traits resemble *P. ridibundus* more than *P. lessonae*, although the hybrids possess two LL and only one R genome. He explained the deviation from the expected dosage effect by “genomic imprinting”, i.e. the overexpression of R and/or repression of L genes in offspring through the maternally inherited R genome. Although being more *P. ridibundus* like would be adaptive for LLR hybrids because it could help them in competition with RR males over access to RR females, the proposed imprinting mechanism cannot work. As [92] pointed out, natural selection cannot act on the LLR hybrids’ R genome, because it is excluded from the germline. Similarly, LRR in our populations cannot become more *P. lessonae* like through natural selection on the L genome, because in this hybrid type, the L is excluded and, hence, an evolutionary dead end. Whatever the true genetic mechanism behind the deviation from dosage ratios (see [92] for alternative explanations), it cannot be denied that water frogs exhibit mosaic-like phenotypes with some traits shaped by genetic information in the double-copy part of the genome (LL) and others by the dominance of the single copy (R).

The results on call differentiation from our study are more in line with those of [23] on morphological characters: the double-genome appears to “pull” the phenotypic expression of the triploid hybrid in the direction of the respective parental species, as expected under the dosage effect hypothesis. This is obvious from the fact call parameter values are either decreasing or increasing in the order LLR-LR-LRR. For two parameters (PPPGR, IPGRIP), however, the dosage effect is skewed in direction of the L-genome, both in the univariate analyses (Figure 4) and the discriminant analysis where along function 1 (mainly representing (IPGRIP and PPPGR) hybrids were located closer to *P. lessonae* than to *P. ridibundus* (Figure 5). This suggests that even in the haploid state the influence by L is stronger than by R. Results from previous studies on *P. esculentus* and another hybridogenetic hybrid, *P. grafi* (a hybrid between the Iberian water frog *P. perezi* and *P. ridibundus* that hemiclonally transmits one copy of *ridibundus* genome), confirm that manifestation of call characteristics in both hybrid lineages converge towards the non-*ridibundus* genome [14]. However, for PGR the opposite was true: all hybrid types resembled *P. ridibundus* more than *P. lessonae*. This variation in the relative “strength” of L and R genomes suggest the existence of additional influences on calls, including the ones discussed below: the breeding system and factors related to geographic distances between populations.

### The role of the breeding system for advertisement call differences

While LR hybrids are considered to be phenotypically intermediate between the parental species LL and RR [12,14] and between their triploid conspecifics LLR and LRR [23], we found considerable variation in the expression of call parameters among LR hybrids from different breeding systems. Compared to mixed ploidy populations, LR hybrids from diploid systems showed higher genetic diversity in the L genome and were less similar to *P. lessonae* in two highly discriminative call parameters (PPPGR and IPGRIP). This difference could possibly be explained by the particularities of genome inheritance in the two population types. LR hybrids from mixed-ploidy populations receive and pass on previously recombined copies of one or both genomes that descended from one diploid and one triploid, or from two triploid parents. LR hybrids from diploid systems, on the other hand, receive the premeiotically excluded genome from a parental species (*P. lessonae* in L-E-systems, *P. ridibundus* in R-E-systems); but they do not transfer it to the next hybrid generation. Because of this “dead end” there is – contrary to mixed ploidy systems - no selection on the “rented” parental L or R genome within the hybrid; selection in diploid systems occurs only in the parental species for which the hybrid’s “interest” is not relevant. The hybrid’s clonally transmitted genome, however, can be the subject of selection processes, if certain hemiclones are more successful than others as suggested by the frozen niche hypothesis [93]. This difference in selective processes suggests that in LR frogs, that exclude the L genome, the R genome may exert a slightly stronger effect on call parameters, with the result that PPPGR and IPGRIP of LR hybrids are slightly higher (and thus more *P. ridibundus* like) than in mixed ploidy systems, although overall they are still more similar to the *P. lessonae* pattern (Figure 4a-e).

Given that previous studies have shown that hybrid and parental females in diploid hybrid systems prefer parental over hybrid males [6,44,45], it would be beneficial for LR hybrid males in diploid systems to sound like the parental species they co-exist and breed with. This seems to be supported by our results from diploid systems. In all three of them, LR hybrids exclude the L and clonally transmit the R genome [55,94]. However, in two of them (Šaštin-Stráže and Herzberg), *P. esculentus* co-occurs with both *P. lessonae* and *P. ridibundus*. Here, the comparatively greater similarity of LR calls to *P. ridibundus* (when compared to those from mixed ploidy systems) may be an adaptation of hybrid males to mimic *P. ridibundus* calls for a reproductive benefit when attempting to mate with *P. ridibundus* females. In fact, for these two populations, low pairwise F_ST_ values between LR and both LL and RR indicate that diploid hybrids are mating with both parental species [55]. In the L-E-system of Hellberg, however, where *P. ridibundus* does not occur and, hence, LR hybrids should mimic *P. lessonae* as much as possible, the shift towards *P. ridibundus* features does not seem to exist. Thus, in all three diploid populations, the basic mechanism is the same, namely selection between different clonal R lineages, but the outcome differs in agreement with the breeding system: it makes the hybrid calls similar to the calls of the parental species that can act as sexual hosts.

### The role of geographic and genetic distance for advertisement call differences

In addition to the marked genotype effect, we also found a population effect on calls, although much smaller and for only two of the five parameters, PPPGR and IPGRIP (Table 3). These two parameters are the same that differentiate best between genotypes and are also influenced by the breeding system. This population effect is not surprising. According to previous studies, *P. esculentus* populations originated from multiple primary hybridization events in sympatric areas of *P. lessonae* and *P. ridibundus* with subsequent dispersal of different hybrid lineages [92,95]. These lineages differ in several ways, including the abilities of the L- and R-genomes to induce and resist genome exclusion [92,96,97], the gamete production patterns and the way how triploids are formed (Pruvost et al., subm.). In light of these genetic differences and the large intraspecific variation in calls of the parental species *P. ridibundus* and *P. lessonae* across Europe [12,35], it seemed plausible to hypothesize that some of the variation among hybrids from different localities has resulted from different call characteristics of the parental haplotypes that were involved in primary hybridizations. This is why we tested for possible spatial and genetic correlations with inter-population call dissimilarities. To avoid mixing populations with different breeding systems and because the number of populations containing only diploid hybrids and those containing LRR hybrids was too low, we restricted the corresponding Mantel tests to a subset of populations containing both LR and LLR individuals. The tests neither revealed a genetic isolation by distance pattern, nor did call differences correlate with geographic distance.

**Figure 7:**
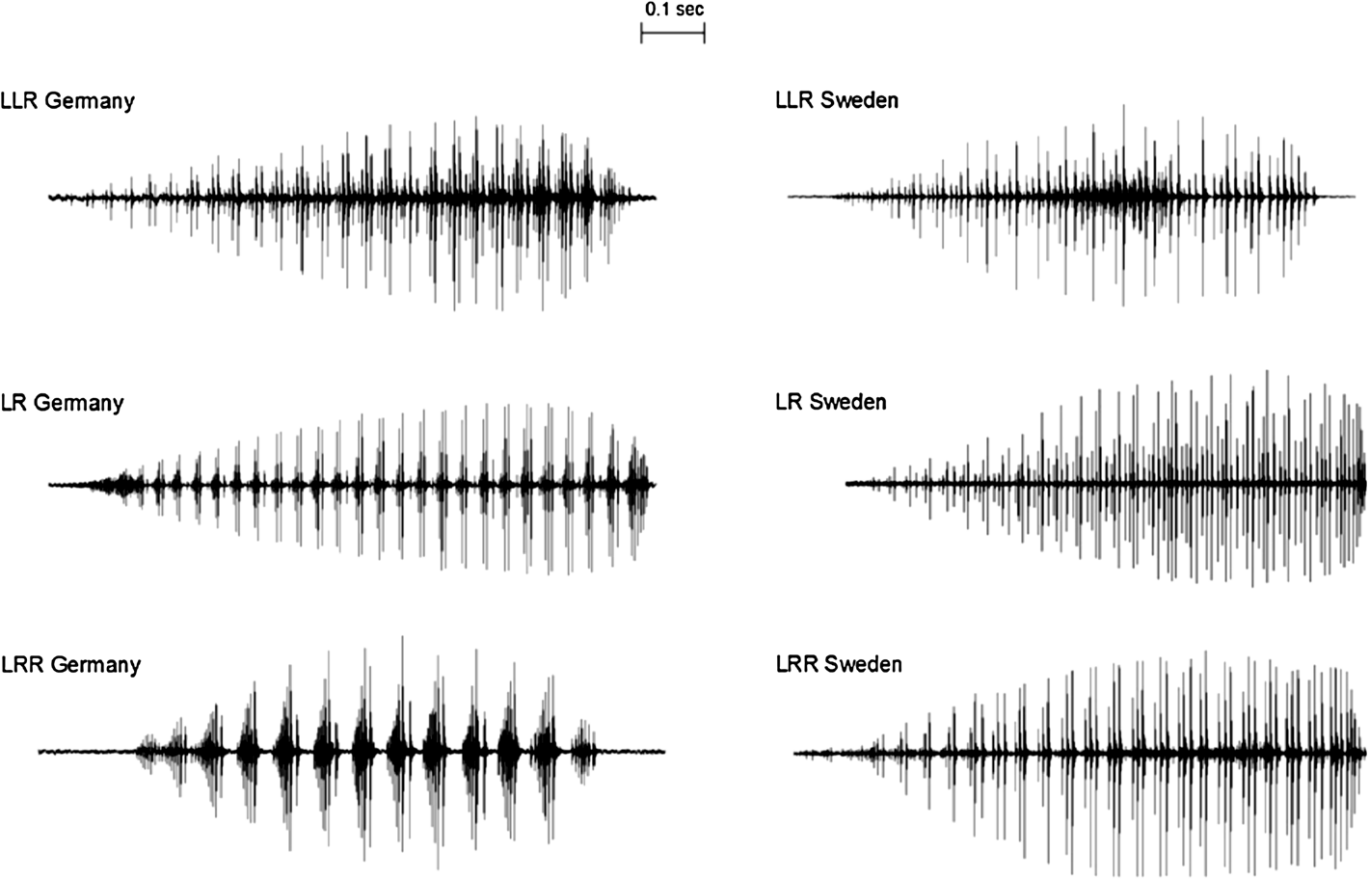
**Comparative overview of advertisement calls from Germany (Döbern) and Sweden (Genarp).** Shown are representative oscillograms of calls by each hybrid type recorded at approximately 20°C.

There was one population in our study that deviated from the general pattern found in the other eight populations. In the northernmost all-hybrid population from Genarp (Southern Sweden), triploid LLR and LRR calls were similar to each other and lay embedded in the same cluster as LR from their own and from other populations (Figure 5). This stood in sharp contrast to the other mixed ploidy populations, e.g. the pond in Döbern (East Germany), where all three hybrid types appeared in different clusters. The difference is also immediately obvious in representative call oscillograms from these two populations: they show clear differences between LLR, LR and LRR for Döbern, but similar patterns for Genarp (Figure 7).

A proximate explanation for the lack of differentiation among calls by frogs from Sweden could be their comparatively low genetic diversity, which has been attributed to their location outside the distribution range of both parental species and close to the northern edge of the Central European distribution range of *P. esculentus*[56,75]. Our results confirm this pattern: among the nine studied populations, *P. esculentus* from Northern Europe had the lowest genetic diversity (He) in the L genome and the second lowest in the R genome. If call differentiation has a genetic basis, a lack thereof among northern European frogs could have several possible explanations. For example, prior to the post-glacial colonization of the north one of the numerous primary hybridizations in Central Europe may have resulted in hybrids that lacked the call differences from the very beginning. Alternatively, a mutation may have disabled the expression of call differences either before or after the colonization. In the absence of parental genotypes, this novel genetic information may have been “frozen” (frozen niche variation model, see [93]) in hybrid lineages that dispersed north. Finally, introgression of nuclear genes from *P. lessonae* into the *P. ridibundus* genome could also have caused a diminution of *ridibundus*-like call characteristics. Nuclear introgression has been found in a number of *P. esculentus* populations [98-101]. Regardless of the exact proximate mechanism, a lack of dosage-specific call differentiation would have gotten established in northern all-hybrid populations if it either turned out neutral (i.e. through genetic drift) or beneficial (e.g. through sexual selection).

An ultimate explanation for the lack of call differentiation in Swedish frogs may lie in the role of male vocalization in female mate choice. From an evolutionary perspective, discrimination of male calls makes sense in diploid populations where hybrid females suffer a severe reproductive disadvantage from mating with hybrid males, since their common offspring are usually unviable due to the accumulation of deleterious alleles in the hybridogenetically transmitted R genome [102,103]. In contrast, genetic fixation of mate preferences in a particular genotype should be impossible in diploid-triploid all-hybrid populations where suitable partners alternate each generation: diploid LR females producing diploid eggs should choose triploid LLR or LRR males; the resulting triploid daughters should choose diploid LR males etc. [66]. Results from playback experiments are consistent with these predictions. *P. esculentus* females from diploid populations prefer calls of *P. lessonae* over those of their own hybrid males [6,44,45]. In contrast, female *P. esculentus* from Sweden did not show any ploidy-specific preference of male advertisement calls [65]. Whether this lack of discrimination reflects that they should not (ultimate reason), or that they cannot differentiate between males of different ploidy, because their calls do not differ much (proximate reason), remains an open question. At present, we also do not know why considerable ploidy-specific call differences do exist in other mixed ploidy hybrid populations (e.g. Döbern), where – from an evolutionary point of view - they also should not play a role in mate choice. Whether in these populations females do use the existing call differences to choose between males of different ploidies remains to be the subject for further studies.

## Conclusions

Across all hybrid types, breeding systems and localities, *P. esculentus* calls are predominantly shaped by the influence of the *lessonae*-genome (L) when it comes to the expression of advertisement call characteristics. However, several genetic particularities – such as genome dosage-sensitive expression in triploids, or more *P. ridibundus*-like call properties due to the “frozen” character of the clonal R genome in diploid systems - provide a perceptible fine tuning of hybrid call manifestation. As there is no rule without exception, we found that genome dosage-sensitive call patterns can be interrupted in certain populations, possibly due to random mutation, introgression or local selective forces. Although we can for now only speculate on the exact mechanisms behind the observed phenomena, our results most certainly confirm that *P. esculentus* populations are a genetically and phenotypically diverse clade. Apart from the evolution of reproductive strategies to gain independence from parental back-crossing, this group has developed regionally variable manifestations of genotype-dependent call differentiation, which could be related to several factors: 1) the reproductive modus responsible for the transmission of the R genome, 2) the features of the original parental genomic heritage and hybrid lineage, 3) the distance from the distribution edge of *P. esculentus* and its two parental species, and 4) the degree to which discrimination between genotypes plays a role in reproductive behaviors. Further studies and carefully designed mate-choice experiments could shed more light on the question whether pronounced call differences among males of different ploidies in most mixed ploidy populations are simply a neutral by-product of allopolyploidy, or could still have a reproductive function, e.g. to facilitate dissortative matings between diploid and triploid hybrids.

## Competing interests

The authors declare that they have no competing interests.

## Authors’ contributions

AH carried out the recording and analysis of calls, analyzed the genetic data and drafted the manuscript. H-UR wrote the proposal on which the study is based and helped in call recording, data statistical analysis and drafting the manuscript. Both authors have read and approved the final manuscript.

## Supplementary Material

Additional file 1 **Microsatellite markers that were originally applied to genotype waterfrog individuals **[58]**,**[75]**,**[104]**-**[106]**.** Click here for file
