# Supplementary material for: Genomic effects on advertisement call structure in diploid and triploid hybrid waterfrogs (Anura, Pelophylax esculentus)
Source: BMC Ecol. 2013 Dec 4;13:47. doi: 10.1186/1472-6785-13-47 (PMC4235041; doi:10.1186/1472-6785-13-47)
Supplement: Additional file 1 — Microsatellite markers that were originally applied to genotype waterfrog individuals [58],[75],[104]-[106]. [file 1472-6785-13-47-S1.doc]

Additional file 1 Microsatellite markers that were originally applied to genotype waterfrog individuals

Not all markers came to use for analysis in this study (see Methods section). The column “dosage” indicates whether a marker could be used to identify triploid hybrids through dosage distribution in the L or R genome.

| **Locus** | **SequenceF** | **SequenceR** | **Reference** | **GenBank No.** | **Use (this study)** | **Dosage** |
| --- | --- | --- | --- | --- | --- | --- |
| CA1b6 | FAM - AAACTCGCGGTTTCCCTTAG | GAGCCAGGTTAAGATAACTGGAG | [75] | EF121548 | L & R | yes |
| Ga1a19 redesigned | FAM - GAC TGG GAG GGA TAG GAA GG | CAG GGG ATT TTC CCA TCA G | [75] | EF121547 | L & R | yes |
| Re1CAGA10 | VIC - CAT GTT TAC CGT CAC TTT AAG AAC AC | CAT CTC TTC AGG TGG CTG GA | [75] | EF121549 | R | No |
| Re2Caga3 | NED - ATG TCG TTA GAG TTC ATA GG | ATC TCA AGT AAT CTG TCT GTC | [75] | EF121550 | R | no |
| ReGA1a23 | NED - ATT GCT TTG GCA GTG AAG G | TGA CAT CAC AGT GGG AGG AG | [58] | EU445523 | L | no |
| Res16 | FAM - GAT CCT GAT TTC CTG CT | GTT TAT TTA CTC TGT TCG TCT T | [104] | AF195843 | not used | yes |
| Res20 | VIC - TTT GTA AAT ATT CCG CTG GTA | CCG AGG TTG GCT GTC ATT A | [104] | AF195845 | L | no |
| Res22 | FAM - ATA CAG GGC TTA GTG AAA TGA A | AAG GGG TTA AAG GTG TGA CTA T | [104] | AF195846 | R | no |
| RlCA18 | FAM - CTC TGC TCC CTC AGC TAT GC | AAA AAG TGG TCC TTT CAT TTT GAG | [105] | AF286386 | L | no |
| RlCA1a27 | PET - GTT CAA GGG GGT CGA AAT AC | CAA ATG GGT CAT CCA CAC C | [58] | EU445522 | L | no |
| RlCA1b5 | NED - CCC AGT GAC AGT GAG TAC CG | CCC AAC TGG AGG ACC AAA AG | [105] | AF286388 | L & R | yes |
| RlCA2a34 | PET - GCT CCA TGC CAA AAG TCT TC | TTG GGT ATG ATA CTA CAA GCT ATG C | [58] | EU445521 | L & R | no |
| RlCA5 | VIC - CTT CCA CTT TGC CCA TCA AG | ATG TGT CGG CAG CTA TGT TC | [105] | AF286385 | not used | no |
| Rrid013A | FAM - CGA GAA TCG AAG TGG AGA GG | ACC CGT CTC CAC AAT ACT GC | [106] | FJ024047 | R | no |
| Rrid059A redesigned | NED - CCC CAT ACA TAT TGT TGG TTC C | ACA CTT ACA CTA AAA AGG ACA TTT ACC | [58] | FJ024048 | R | no |
| Rrid064A | PET - TGT ACG GGC CTT TAG ACT GG | AAC TTT TTG AAG GCC CCT TG | [58] | EU445524 | R | no |
| Rrid135A | NED - TCT TTT GTT TTA GCG CAC CT | CTG CCC GTC TAA GCA AGT GT | [58] | EU445526 | R | no |
| Rrid169A | VIC - CGG AAC TCC GCT TTA ATC AC | CCC ATG TTG TCG TTG AGC TA | [58] | EU445525 | R | no |
